# The relationship between thyroid function and cerebral blood flow in mild cognitive impairment and Alzheimer’s disease

**DOI:** 10.1371/journal.pone.0214676

**Published:** 2019-04-03

**Authors:** Shohei Nomoto, Ryuta Kinno, Hirotaka Ochiai, Satomi Kubota, Yukiko Mori, Akinori Futamura, Azusa Sugimoto, Takeshi Kuroda, Satoshi Yano, Hidetomo Murakami, Takako Shirasawa, Takahiko Yoshimoto, Akira Minoura, Akatsuki Kokaze, Kenjiro Ono

**Affiliations:** 1 Division of Neurology, Department of Medicine, Showa University School of Medicine, Tokyo, Japan; 2 Department of Hygiene, Public Health and Preventive Medicine, Showa University School of Medicine, Tokyo, Japan; Nathan S. Kline Institute, UNITED STATES

## Abstract

The thyroid hormones have been reported to be associated with cognitive decline and Alzheimer’s disease. The relationship between thyroid function within the normal range and cerebral blood flow in Alzheimer’s disease patients has been shown in a recent study. Mild cognitive impairment is often the first stage of Alzheimer’s disease; thus, early diagnosis is important. The present study investigated the relationship between thyroid function and regional cerebral blood flow in patients with mild cognitive impairment and Alzheimer’s disease. A total of 122 memory clinic outpatients who underwent thyroid function testing and single photon emission computed tomography were divided into mild cognitive impairment, Alzheimer’s disease, and Normal groups. Regional cerebral blood flow was calculated using a three-dimensional stereotactic region of interest template in an automated cerebral perfusion single photon emission computed tomography analysis system. Multiple regression analysis adjusted for age and sex was conducted to examine the relationships between thyroid hormones and regional cerebral blood flow. Thyroid stimulating hormone was significantly associated with regional cerebral blood flow in the bilateral temporal, bilateral pericallosal, and bilateral hippocampal regions in the mild cognitive impairment group. In the Alzheimer’s disease group, free triiodothyronine was significantly associated with regional cerebral blood flow in the bilateral parietal, right temporal, and bilateral pericallosal regions. The present study showed the association of thyroid stimulating hormone with regional cerebral blood flow in the mild cognitive impairment group and the association of free triiodothyronine with regional cerebral blood flow in the Alzheimer’s disease group. These study findings could contribute to the early diagnosis of mild cognitive impairment at general memory clinics and the prevention of subsequent progression to Alzheimer’s disease.

## Introduction

The thyroid hormones have been reported to be associated with cognitive decline and Alzheimer’s disease (AD). A total of 14 of 23 studies demonstrated a correlation between subclinical hypothyroidism (SCH) and cognitive function [1], while an association between thyroid stimulating hormone (TSH) levels within the normal range and the risk of AD has also been reported [2]. A single photon emission computed tomography (SPECT) study in AD patients showed decreased regional cerebral blood flow (rCBF) in the posterior cingulate and parietotemporal association cortices [3]. Kimura et al. identified a significant inverse correlation between TSH levels within the normal range and rCBF in the right middle and inferior temporal regions in AD patients [4]. Meanwhile, Chiaravalloti et al. demonstrated a positive correlation between TSH levels within the normal range and cortical glucose consumption in the bilateral anterior cingulate cortices and left frontal lobe [5]. Furthermore, Haji et al. reported significant decreases in rCBF localized in the temporal lobe and thalamus in AD patients with SCH [6]. However, the mechanism of the association between thyroid function and rCBF in the area related to memory seems not to have been elucidated.

MCI is often the first stage of AD, with a rate of progression to dementia of 7% per year [7]; therefore, early diagnosis is important. Several studies have identified decreased blood flow in the temporal lobe as a characteristic of MCI with increased risk of progression to AD [8, 9]. Although the association between TSH and MCI in women has been observed [10], many other studies have reported no significant correlation between thyroid hormone levels and the risk of MCI [11–13], and a consensus has yet to be reached. To the best of our knowledge, the relationship between CBF and thyroid hormones has not been investigated in MCI patients.

The present study investigated the relationship between thyroid function and rCBF in patients with MCI and AD in order to elucidate the pathological effects of thyroid hormones in these neurological disorders. Measurement of the levels of these hormones may form the basis for early diagnosis in general memory clinics.

## Materials and methods

### Participants

The participants in this study were outpatients examined at the memory clinic of the Division of Neurology in the Department of Medicine at the Showa University School of Medicine between April 2016 and March 2018 who underwent thyroid function testing and SPECT. Patients were divided into MCI, AD, and Normal groups based on the National Institute on Aging-Alzheimer’s Association (NIA-AA) diagnostic criteria [14, 15].

Since the objective was to investigate whether fluctuations in thyroid hormone levels within the normal range affect AD pathology, patients with thyroid hormone levels outside the normal range or with a clinical history of thyroid disease were excluded. Also excluded were patients with other forms of cognitive dysfunction, such as: vascular-type dementia or dementia with Lewy bodies; cerebrovascular disease; psychiatric disorder such as major depressive disorder or psychosis; severe head trauma; alcohol dependency; severe heart disease; refractory diabetes mellitus; kidney or liver failure; or severe anemia. In addition, patients with non-age-related white matter lesions on magnetic resonance imaging (MRI) were excluded.

Routine clinical examination comprised blood counts, biochemical assays, and measurement of thyroid hormone and vitamin levels. All patients underwent MRI and SPECT. Age, sex, and Hasegawa Dementia Scale-Revised (HDS-R) and Mini Mental State Examination-Japanese (MMSE-J) score data were obtained from patient medical records.

All 122 outpatients participated in this study. After exclusions, 96 patients (34 men, 62 women; median age 81 years; age range 50–97 years) remained for analysis (All relevant data are listed in S1 Table). Written, informed consent was obtained from participants if they were mentally competent from consultation, HDS-R and MMSE-J, if not, informed consent was obtained from their closest relative at the time of the initial visit. The study and its consent procedure were approved by the Medical Ethics Committee of Showa University School of Medicine (approval no. 2445, November 19, 2018). Levels of free triiodothyronine (fT3; normal range, 1.71–3.71 pg/mL), free thyroxine (fT4; normal range, 0.70–1.48 ng/dL), and TSH (normal range, 0.35–4.94 μIU/mL) were analyzed using a chemiluminescent microparticle immunoassay (Architect i1000SR; Abbott Japan, Tokyo, Japan).

### SPECT acquisition

SPECT was performed with patients lying in a supine position. Images were acquired using a double-detector gamma camera system (Discovery NM/CT 670; GE Healthcare, Tokyo, Japan) after administration of a 600 MBq technetium-99mTc ethyl cysteinate dimer bolus. Non-invasive measurement of global CBF was performed using a Patlak plot [16]. Regional CBF was calculated using a three-dimensional stereotactic region of interest (ROI) template (3DSRT) [17], which produces anatomically standardized images [18], in an automated cerebral perfusion SPECT analysis system. Based on statistical parametric mapping, this program uses 3DSRT to calculate rCBF. A total of 636 ROIs were classified into 12 areas (callosomarginal region, precentral region, central region, parietal region, angular gyrus, temporal region, posterior region, pericallosal region, lenticular nucleus, thalamus, hippocampus, and cerebellar hemisphere). Statistical Parametric Mapping (SPM) performs statistical analysis by comparison with healthy databases. However, preclinical AD is probably included in the healthy databases in SPM. Thus, statistical analysis using that database is not suitable when targeting the Normal group who visited a memory clinic. Therefore, 3DSRT, which carries out processing by quantitative analysis, was used. [19]

Of these 12 areas, five (parietal region, temporal region, pericallosal region, thalamus, and hippocampus) were used in this study, because rCBF values in the parietal, pericallosal, and hippocampal regions are clinically related to MCI and AD, and rCBF values in the temporal region and thalamus are reportedly significantly decreased in AD patients with SCH [6].

### Statistical analysis

Data are presented as medians (25th percentile, 75th percentile) for continuous variables or n (%) for categorical variables. The Kruskal-Wallis test or the chi-squared test was used to compare characteristics among the Normal, MCI, and AD groups. Spearman’s correlation coefficients between thyroid hormone (fT3, fT4, or TSH) levels and rCBF in each ROI were calculated for each group (Normal, MCI, and AD). A multiple linear regression model was used to evaluate the relationships between thyroid hormone levels and rCBF. Age and sex were adjusted for in the model [20]. Therefore, the multiple linear regression model included rCBF as a dependent variable and the other variables (age, sex, each thyroid hormone) as independent variables. The goodness of fit of the model was evaluated by the coefficient of determination (R^2^). The presence of multicollinearity in multivariate models was evaluated using the variance inflation factor, with a value >5 suggesting its presence. An age-matched group was also created and analyzed statistically in the same way. Non-normally distributed variables were entered into the model after logarithmic transformations. A p-value < 0.05 was considered significant. Statistical analyses were performed using the software package JMP version 13.0.0.

## Results

### Characteristics of each group

Table 1 shows the patients’ demographic and clinical characteristics together with serum TSH, fT3, and fT4 levels and rCBF values in the bilateral parietal, temporal, pericallosal, thalamic, and hippocampal regions for each group. Significant intergroup differences were observed in age, HDS-R and MMSE scores, fT3, and rCBF values in the right parietal, bilateral temporal, bilateral pericallosal, bilateral thalamic, and bilateral hippocampal regions. No significant differences were observed with regard to sex, fT4, TSH, and rCBF in the left parietal region. Among MCI patients, 27 were amnestic type, of which 8 were single domain and 19 were multiple domain, and 4 were non-amnestic type, of which 2 were single domain and 2 were multiple domain. An age-matched group was also created, but when it was strictly matched, there were only 5 cases each. Therefore, matching was performed within 3 years of age, and each group then had 15 cases. Statistical analysis was carried out of those groups, and significant intergroup differences were observed in rCBF values in the left parietal and bilateral hippocampal regions (data not shown).

**Table 1:** Comparisons of characteristics among the Normal, MCI, and AD groups.

Characteristics		Normal (n = 17)	MCI (n = 31)	AD (n = 48)	p value*
Age (y)		74.0 (69.5, 78.0)	81.0 (72.0, 85.0)	82.5 (77.0, 87.0)	0.001
Men, n (%)		5 (29.4)	13 (41.9)	16 (33.3)	0.627
HDS-R score		29.0 (27.0, 30.0)	26.0 (23.0, 27.0)	15.5 (13.0, 21.8)	<0.001
MMSE score		29.0 (27.8, 30.0)	25.0 (24.0, 28.0)	19.0 (15.3, 22.0)	<0.001
fT3 (pg/mL)		2.66 (2.40, 2.80)	2.50 (2.28, 2.67)	2.40 (2.22, 2.64)	0.028
fT4 (ng/mL)		1.00 (0.91, 1.13)	0.98 (0.92, 1.04)	0.97 (0.89, 1.05)	0.412
TSH (μIU/mL)		1.36 (1.01, 1.91)	1.58 (1.04, 2.32)	1.68 (1.07, 2.30)	0.664
rCBF (mL/100 g/min)					
Parietal	Left	36.05 (34.12, 40.59)	34.23 (31.40, 37.44)	34.00 (30.23, 36.95)	0.061
	Right	35.86 (34.05, 42.40)	33.70 (31.14, 38.21)	34.10 (30.38, 36.68)	0.045
Temporal	Left	37.86 (35.08, 41.78)	34.50 (31.39, 36.76)	34.34 (31.04, 37.68)	0.011
	Right	37.15 (34.48, 41.45)	35.22 (31.29, 37.21)	32.90 (30.35, 37.39)	0.005
Pericallosal	Left	42.88 (40.02, 50.22)	41.80 (37.41, 45.02)	39.90 (36.65, 44.66)	0.043
	Right	42.90 (40.09, 49.35)	41.58 (37.41, 44.00)	39.77 (36.66, 43.90)	0.028
Thalamus	Left	46.11 (43.33, 54.64)	43.68 (36.03, 49.01)	40.12 (37.31, 47.40)	0.005
	Right	46.78 (41.98, 56.69)	44.23 (38.32, 48.36)	42.11 (37.57, 46.65)	0.017
Hippocampus	Left	36.73 (34.74, 40.31)	33.34 (29.61, 38.69)	31.29 (28.39, 34.29)	<0.001
	Right	37.62 (32.25, 41.42)	31.24 (29.14, 35.52)	30.20 (27.64, 33.57)	<0.001

MCI, mild cognitive impairment; AD, Alzheimer’s-type dementia; HDS-R, Hasegawa Dementia Scale-Revised; MMSE, Mini-mental State Examination; fT3, free triiodothyronine; fT4, free thyroxine; TSH, thyroid stimulating hormone; rCBF, regional cerebral blood flow

Data are presented as medians (25, 75 percentiles) or n (%).

*Kruskal-Wallis or chi-squared test

### Correlations of thyroid hormone levels

Table 2 shows the correlations between thyroid hormone levels and rCBF in each of the 10 analyzed regions. In the Normal group, although there was no significant difference, rCBF in all areas showed a positive correlation with fT3 and a negative correlation with TSH. In the MCI group, significant correlations were observed between TSH and rCBF values in the bilateral pericallosal, right thalamic, and left hippocampal regions. In the AD group, significant correlations were observed between fT3 and rCBF values in the bilateral parietal, right temporal, and right pericallosal regions. In the groups matched within 3 years of age, significant correlations were observed between fT4 and rCBF values in the left parietal and right thalamic regions in the AD group, whereas no significant correlations were observed in the Normal group and the MCI group (data not shown).

**Table 2:** Correlations of rCBFs with fT3, fT4, and TSH in each group.

			fT3	fT4	TSH
	rCBFs		ρ	ρ	ρ
Normal group	Parietal	Left	0.321	0.186	-0.378
		Right	0.322	0.136	-0.365
	Temporal	Left	0.195	0.108	-0.400
		Right	0.144	-0.032	-0.262
	Pericallosal	Left	0.378	0.135	-0.397
		Right	0.385	0.123	-0.395
	Thalamus	Left	0.318	-0.098	-0.302
		Right	0.128	0.049	-0.289
	Hippocampus	Left	0.480	0.213	-0.338
		Right	0.304	-0.029	-0.304
MCI group	Parietal	Left	-0.173	-0.048	0.209
		Right	-0.217	0.088	0.239
	Temporal	Left	-0.047	-0.119	0.244
		Right	-0.074	-0.080	0.279
	Pericallosal	Left	-0.277	-0.219	0.383*
		Right	-0.307	-0.298	0.384*
	Thalamus	Left	-0.180	-0.200	0.164
		Right	-0.167	-0.291	0.359*
	Hippocampus	Left	-0.079	-0.218	0.377*
		Right	-0.140	-0.117	0.345
AD group	Parietal	Left	0.296*	0.026	0.112
		Right	0.383*	-0.067	0.021
	Temporal	Left	0.193	-0.052	0.043
		Right	0.298*	-0.079	-0.066
	Pericallosal	Left	0.264	0.023	0.059
		Right	0.341*	0.053	-0.002
	Thalamus	Left	0.278	0.020	-0.166
		Right	0.186	-0.093	-0.140
	Hippocampus	Left	0.168	0.038	-0.058
		Right	0.213	0.020	-0.169

rCBF, regional cerebral blood flow; fT3, free triiodothyronine

fT4, free thyroxine; TSH, thyroid stimulating hormone

ρ: Spearman’s correlation coefficients.

*p<0.05

### Multiple linear regression analysis adjusted for age and sex

Tables 3–5 show the associations between thyroid hormones and rCBF by multiple linear regression analysis adjusted for age and sex in each region for each group. In the Normal group, although there was no significant difference, rCBF in all areas showed a negative correlation with TSH. In the MCI group, significant correlations were observed between TSH and rCBF values in the bilateral temporal, bilateral pericallosal, and bilateral hippocampal regions. In the AD group, significant correlations were observed between fT3 and rCBF values in the bilateral parietal, right temporal, and bilateral pericallosal regions. On statistical analysis of the groups matched within 3 years of age, no significant correlations were observed (data not shown).

**Table 3:** Associations between rCBFs and thyroid hormones in the Normal group.

rCBFs	Thyroid hormones	B	Standard error	p value	R^2^ (adjusted R^2^)
Parietal					
Left	fT3	0.174	0.385	0.659	0.328(0.173)
	fT4	-0.127	0.235	0.598	0.333(0.179)
	TSH	-0.080	0.070	0.274	0.380(0.237)
Right	fT3	0.057	0.426	0.895	0.282(0.116)
	fT4	-0.159	0.257	0.547	0.301(0.140)
	TSH	-0.095	0.077	0.236	0.357(0.209)
Temporal					
Left	fT3	-0.089	0.412	0.833	0.318(0.161)
	fT4	-0.179	0.248	0.483	0.342(0.190)
	TSH	-0.049	0.077	0.541	0.336(0.182)
Right	fT3	-0.164	0.398	0.688	0.309(0.150)
	fT4	-0.231	0.237	0.347	0.349(0.198)
	TSH	-0.033	0.075	0.668	0.311(0.152)
Pericallosal					
Left	fT3	0.183	0.420	0.671	0.467(0.343)
	fT4	-0.201	0.253	0.441	0.484(0.365)
	TSH	-0.079	0.077	0.326	0.499(0.383)
Right	fT3	0.167	0.433	0.706	0.416(0.282)
	fT4	-0.208	0.261	0.439	0.437(0.307)
	TSH	-0.077	0.080	0.350	0.449(0.322)
Thalamus					
Left	fT3	0.090	0.401	0.827	0.440(0.310)
	fT4	-0.255	0.236	0.299	0.484(0.365)
	TSH	-0.021	0.076	0.791	0.441(0.312)
Right	fT3	-0.256	0.451	0.580	0.402(0.264)
	fT4	-0.072	0.279	0.801	0.390(0.249)
	TSH	-0.037	0.086	0.674	0.396(0.256)
Hippocampus					
Left	fT3	0.383	0.408	0.365	0.428(0.297)
	fT4	-0.065	0.258	0.805	0.393(0.252)
	TSH	-0.021	0.080	0.797	0.393(0.253)
Right	fT3	0.062	0.414	0.884	0.467(0.344)
	fT4	-0.278	0.242	0.272	0.515(0.403)
	TSH	-0.028	0.078	0.728	0.471(0.349)

rCBF, regional cerebral blood flow; fT3, free triiodothyronine; fT4, free thyroxine; TSH, thyroid stimulating hormone

B(unstandardized coefficient) is shown after adjustment for age and sex in the multiple linear regression model.

Non-normally distributed variables were entered into the model after logarithmic transformations.

**Table 4:** Associations between rCBFs and thyroid hormones in the MCI group.

rCBFs	Thyroid hormones	B	Standard error	p value	R^2^ (adjusted R^2^)
Parietal					
Left	fT3	-0.180	0.200	0.378	0.220(0.134)
	fT4	0.018	0.236	0.939	0.197(0.108)
	TSH	0.055	0.042	0.201	0.245(0.161)
Right	fT3	-0.262	0.220	0.244	0.181(0.090)
	fT4	0.037	0.262	0.890	0.139(0.043)
	TSH	0.080	0.045	0.086	0.229(0.143)
Temporal					
Left	fT3	0.007	0.182	0.970	0.372(0.302)
	fT4	-0.177	0.208	0.403	0.388(0.320)
	TSH	0.074	0.036	0.048	0.458(0.397)
Right	fT3	0.000	0.204	0.999	0.326(0.251)
	fT4	-0.131	0.236	0.584	0.334(0.260)
	TSH	0.094	0.039	0.024	0.444(0.382)
Pericallosal					
Left	fT3	-0.268	0.209	0.211	0.287(0.208)
	fT4	-0.265	0.245	0.288	0.275(0.195)
	TSH	0.101	0.041	0.020	0.383(0.315)
Right	fT3	-0.302	0.220	0.182	0.223(0.137)
	fT4	-0.331	0.257	0.208	0.217(0.130)
	TSH	0.107	0.043	0.020	0.322(0.247)
Thalamus					
Left	fT3	-0.200	0.251	0.432	0.348(0.276)
	fT4	0.000	0.295	0.999	0.333(0.259)
	TSH	0.032	0.053	0.551	0.342(0.269)
Right	fT3	-0.181	0.264	0.500	0.301(0.223)
	fT4	-0.358	0.302	0.246	0.324(0.249)
	TSH	0.094	0.053	0.089	0.362(0.292)
Hippocampus					
Left	fT3	-0.083	0.205	0.687	0.424(0.360)
	fT4	-0.246	0.234	0.301	0.443(0.382)
	TSH	0.104	0.038	0.012	0.545(0.494)
Right	fT3	-0.172	0.226	0.453	0.348(0.276)
	fT4	-0.243	0.262	0.362	0.355(0.283)
	TSH	0.103	0.044	0.027	0.447(0.385)

rCBF, regional cerebral blood flow; fT3, free triiodothyronine; fT4, free thyroxine; TSH, thyroid stimulating hormone

B(unstandardized coefficient) is shown after adjustment for age and sex in the multiple linear regression model.

Non-normally distributed variables were entered into the model after logarithmic transformations.

**Table 5:** Associations between rCBFs and thyroid hormones in the AD group.

rCBFs	Thyroid hormones	B	Standard error	p value	R^2^ (adjusted R^2^)
Parietal					
Left	fT3	0.462	0.170	0.009	0.169(0.112)
	fT4	-0.037	0.197	0.850	0.030(-0.036)
	TSH	0.016	0.048	0.739	0.032(-0.034)
Right	fT3	0.613	0.173	0.001	0.235(0.183)
	fT4	-0.106	0.209	0.615	0.022(-0.045)
	TSH	-0.012	0.051	0.817	0.018(-0.049)
Temporal					
Left	fT3	0.225	0.156	0.158	0.068(0.004)
	fT4	-0.096	0.171	0.575	0.031(-0.035)
	TSH	0.024	0.042	0.566	0.031(-0.035)
Right	fT3	0.383	0.157	0.019	0.137(0.078)
	fT4	-0.072	0.179	0.689	0.024(-0.043)
	TSH	-0.006	0.044	0.885	0.021(-0.046)
Pericallosal					
Left	fT3	0.395	0.181	0.034	0.139(0.081)
	fT4	-0.027	0.204	0.894	0.046(-0.019)
	TSH	0.026	0.050	0.605	0.051(-0.013)
Right	fT3	0.453	0.169	0.010	0.164(0.107)
	fT4	-0.014	0.195	0.941	0.028(-0.038)
	TSH	-0.004	0.048	0.928	0.028(-0.038)
Thalamus					
Left	fT3	0.339	0.176	0.060	0.177(0.120)
	fT4	-0.040	0.196	0.839	0.108(0.047)
	TSH	-0.019	0.048	0.693	0.110(0.050)
Right	fT3	0.216	0.190	0.264	0.086(0.024)
	fT4	-0.182	0.205	0.379	0.076(0.013)
	TSH	-0.033	0.050	0.518	0.068(0.005)
Hippocampus					
Left	fT3	0.242	0.162	0.142	0.142(0.083)
	fT4	0.096	0.177	0.590	0.104(0.043)
	TSH	-0.002	0.043	0.957	0.098(0.037)
Right	fT3	0.237	0.167	0.162	0.133(0.074)
	fT4	0.032	0.182	0.863	0.094(0.032)
	TSH	-0.034	0.044	0.448	0.105(0.044)

rCBF, regional cerebral blood flow; fT3, free triiodothyronine; fT4, free thyroxine; TSH, thyroid stimulating hormone

B(unstandardized coefficient) is shown after adjustment for age and sex in the multiple linear regression model.

Non-normally distributed variables were entered into the model after logarithmic transformations.

## Discussion

The present study investigated the effects of fluctuations in thyroid hormone levels within the normal range on cerebral blood flow in MCI and AD patients. On 3DSRT analysis, significantly lower rCBF values were observed in the right temporal, bilateral pericallosal, bilateral thalamic, and left hippocampal regions in the AD group than in the Normal group and also in the left temporal and right hippocampal regions in both the MCI and AD groups than in the Normal group. These findings are consistent with the SPECT patterns previously reported in AD patients [21]. Regarding the relationship between thyroid function and rCBF in each ROI, significant correlations were observed for TSH with rCBF in the bilateral pericallosal, right thalamic, and left hippocampal regions in the MCI group and for fT3 with rCBF in the bilateral parietal, right temporal, and right pericallosal regions in the AD group. On multiple regression analysis adjusted for age on initial examination and sex regarding the correlation between fT3, fT4, and TSH levels and rCBF in each ROI for each group, no significant correlations were observed in the Normal group, but rCBF in all areas showed a negative correlation with TSH. Significant correlations were observed between TSH and rCBF in the bilateral temporal, bilateral pericallosal, and bilateral hippocampal regions in the MCI group. Significant correlations were also observed between fT3 and rCBF in the bilateral parietal, right temporal, and bilateral pericallosal regions in the AD group. The present findings suggest that TSH and fT3 may be related to cerebral function in the MCI and AD stages of dementia, respectively.

TSH was correlated with cerebral function in the MCI stage, but this relationship was not observed in the AD stage. One model of dynamic biomarkers reflecting the progression of AD describes accumulation of β-amyloid leading to synaptic dysfunction, tau-related neurodegeneration, changes in brain structure, and, ultimately, cognitive dysfunction [22]. Meanwhile, thyroid function may also be involved in aspects of AD pathology, including β-amyloid deposition and neuronal apoptosis, with abnormal TSH levels potentially promoting phosphorylation of tau proteins, which are associated with AD pathogenesis [23, 24]. Ojala et al. reported better cognitive function in association with higher levels of TSH, although the difference was not significant [25]. Another study by Annerbo et al. suggested that lower TSH levels are associated with a higher risk of progression from MCI to AD [26]. These findings suggest that, at the stage of MCI, when TSH becomes high as a compensatory effect for cognitive decline, it may be correlated with rCBF, one of the brain function indicators. According to Yong-Hong, AD patients show abnormalities of the hypothalamic-pituitary-thyroid axis and in its biofeedback [27]. Thomas et al. reported that patients with AD show a blunted TSH response to thyrotropin-releasing hormone (TRH) [28]. Luo suggested that, in the hippocampus of AD patients, TRH is lower than in controls [23]. These factors may be among the causes of the loss of the relationship between TSH and rCBF in AD. Although the specific mechanism could not be investigated, these factors may be related in a complex manner, and these findings were obtained. The present analysis of thyroid hormones and rCBF in AD demonstrated a significant correlation between fT3 and rCBF in the bilateral parietal, right temporal, and right pericallosal regions. These findings differ from those obtained by Kimura et al. using FineSRT [4]. Regarding the relationship between AD and thyroid function, Prinz et al. reported that a study on thyroid function and cognitive function in healthy individuals showed a relationship with fT4 in particular [29]. In addition, a prospective study by de Jong et al. suggested a correlation between fT4 and medial temporal lobe atrophy [30]. Conversely, Thomas et al. reported a significant difference in fT3 levels between severe AD patients and healthy controls [28]. In addition, according to a study by Davis et al., though T3 concentrations in the prefrontal cortex were not significantly different between Braak I–II brains and controls, in Braak V–VI brains, T3 concentrations were significantly lower than in controls [24]. From these studies, fT3 and rCBF may be related in severe AD patients. The significant correlation between fT3 and rCBF observed in the present AD group may be because this group included few patients with comparatively mild AD patients compared with other studies due to the establishment of a separate MCI group. In the present study, in the AD group compared with the Normal group, fT3 was significantly lower, consistent with this. In addition, this may be related to the result of the correlation between fT4 and rCBF in the AD group in the analysis of the groups matched within 3 years of age, which included relatively young participants. Further research regarding the relationship between rCBF and fT3 and fT4 in AD patients is required.

Although it was not significant, there was a negative correlation between TSH and rCBF in the Normal group on multiple linear regression analysis adjusted for age and sex. From the studies of Marangell et al., serum TSH was inversely related to both global CBF and rCBF [31]. This study suggested that, because the inverse correlation may also be found with TSH within the normal range, an inverse correlation of TSH and rCBF in all regions in the Normal group was observed. Meanwhile, global CBF in hypothyroidism could be decreased due to increased vascular resistance or decreased cardiac output, rather than decreased brain function [32, 33]. With TSH in the normal range, the effect on rCBF may be weak compared to the effect of pathological change due to AD, so an inverse correlation of TSH within the normal range, and rCBF in the MCI and AD group may not be observed.

The present study has several limitations. Since TRH concentrations were not measured in this study, the above could not be confirmed. The MCI and AD classifications were based on the NIA-AA diagnostic criteria; however, biomarkers related to β-amyloid were not measured. Although there is reportedly no significant difference between the precision of SPECT and that of positron emission tomography in the NIA-AA diagnostic criteria [34], SPECT is not listed as a biomarker. Therefore, strict diagnoses of MCI and AD including biomarkers were not made. Similarly, preclinical AD was also not diagnosed. Further study with a larger patient population is required to directly confirm our hypothesis that there are various relationships of rCBF with TSH in the MCI stage and with fT3 in the AD stage.

## Conclusion

The present study showed the association of TSH with rCBF in the MCI group and the association of fT3 with rCBF in the AD group. These study findings could contribute to the early diagnosis of MCI in general memory clinics and the prevention of subsequent progression to AD.

## Supporting information

S1 Table Patient characteristics and all their relevant data. (XLSX)
